# Detrimental Effects of Induced Soil Compaction on Morphological Adaptation and Physiological Plasticity of Selected Multipurpose Tree Species

**DOI:** 10.3390/plants12132468

**Published:** 2023-06-28

**Authors:** Muhammad Asif, Muhammad Farrakh Nawaz, Irfan Ahmad, Muhammad Haroon U. Rashid, Taimoor Hassan Farooq, Muhammad Kashif, Sadaf Gul, Qian Li

**Affiliations:** 1 Department of Forestry and Range Management, University of Agriculture, Faisalabad 38000, Pakistan; asif.chohan@yahoo.com (M.A.); kf_uaf@yahoo.com (M.F.N.); irfanahmad@uaf.edu.pk (I.A.); 2 Institute of Environmental Studies, University of Karachi, Karachi 75270, Pakistan; 3 Bangor College China, A Joint Unit of Bangor University and Central South University of Forestry and Technology, Changsha 410004, China; t.farooq@bangor.ac.uk; 4 Department of Mathematics and Statistics, University of Agriculture, Faisalabad 38000, Pakistan; mkashif@uaf.edu.pk; 5 Department of Botany, University of Karachi, Karachi 75270, Pakistan; sadafgpk@yahoo.com; 6 College of Forestry, Central South University of Forestry and Technology, Changsha 410004, China

**Keywords:** soil bulk density, heavy machinery, morphological traits, physiological traits, agro-forest trees

## Abstract

Soil compaction has become a global problem affecting soil worldwide. With an increased population, more demands for food and wood have resulted in intensive cultivation and increased mechanization of our farmlands and irrigated plantations. The use of heavy machinery results in soil compaction, which affects the entire soil ecosystem. This study was conducted to analyze the impact of compacted soil on germination and initial growth stages of four major agro-forest trees of central Punjab, Pakistan. Morpho-physiological traits of all selected species (*Eucalyptus camaldulensis, Albizia lebbeck, Vachellia nilotica*, and *Zyziphus mauritiana*) were measured against soil compaction. Results indicated that the root and shoot length, biomass, root–shoot ratio, diameter at root collar, no. of leaves and branches, leaf area, germination, and survival %, and physiological traits (i.e., photosynthetic rate, transpiration rate, stomatal conductance, internal CO_2_ concentration, and photosynthetic water use efficiency) were significantly affected by the induced soil compaction. *Eucalyptus camaldulensis* Dehnh. performed better and exhibited 96% germination percentage under (1.40 mg m^−3^) compaction level and gradually decreased by 11% with the increase of compaction level (1.80 mg m^−3^). It shows that the shorter roots developed due to soil compaction decreased water use efficiency, photosynthesis, and whole-plant physiological performance. The findings concluded that judicious use of machinery is highly desired for sustainable and good-quality wood production from farm trees.

## 1. Introduction

Agriculture is the basis of the economy for many developing nations like Pakistan. The diverse climatic conditions of Pakistan are not only suitable for agriculture but also conducive for forestry and agro-forestry [1]. Soil is one of the most important factors as far as agriculture and forestry are concerned. Soil quality plays a critical role in the progress and survival of societies in a specific region [2]. It provides the necessities of life and other valuable products to human beings [3]. To handle universal issues like food shortage, environmental and climatic instability, and energy and water crisis, the sustainable use of soil is essential. However, intensive cultivation has increased the mechanization of our farmlands and irrigated plantations [4]. This increased use of machinery for different operations in agriculture and forestry has resulted in soil compaction [5,6]. The use of heavy machinery in forest operations has several advantages, including increased productivity and efficiency [6]. On the other hand, the major problem is the compaction of soil, i.e., a rise in soil parts per unit of volume [7,8]. Compaction of soil is the process by which soil bulk density is increased, and soil particles/grains become compacted, resulting in closer interaction with each other and reduced porosity and volume resulting in limited root penetration, poor soil infiltration, and drainage [9,10].

The compaction of soil has become a global issue. Soil compaction changes the physical soil structure, affecting the chemical properties of soil, soil microorganisms, plant growth, and its development [11,12]. Nowadays, forestry operations in all developed countries are carried out by using vehicular machines. This vehicular intervention causes soil compaction and affects physical and chemical soil quality in the arable operation [8]. The systematization of farms and forest areas has become a crucial community need. This includes intensive cropping patterns that directly or indirectly affect soil structure and ultimately result in soil degradation [13,14]. An area of about 32 million hectares in Europe, 17 million hectares in Africa, 10 million hectares in Asia, 4 million hectares in Australia, and a few parts of Northern America has been estimated as affected by soil compaction [11,15].

Many studies have been conducted to study soil compaction-tolerant crop plants, but studies reporting soil compaction tolerance of trees are scarce [16]. Most reviews investigated the undesirable impacts of compaction on crops and grazing areas [5]. Similar impacts were found in the woodlands of pine species that were subjected to heavy vehicular traffic during felling and other silvicultural operations [17]. Generally, soil compaction adversely affects seedling emergencies [9], limits shoot development, and stunts root growth [6]. Soil compaction negatively affects soil surface quality, and rare cases have been reported where the slight removal of the upper layer of compacted soil was advantageous for coarse particle soils [18]. It is also worth mentioning that the major needs for industrial wood (72%) and fuelwood (90%) in Pakistan are fulfilled by the wood coming from farmlands [19]. So, in the future, the proposed increased use of machinery for cultivation in Pakistan can drastically affect trees growing on farmlands.

Agro-forestry systems based on *Eucalyptus camaldulensis, Albizia lebbeck, Vachellia nilotica*, and *Zyziphus mauritiana* are sustainable alternative land-use systems for improving soil structure and obtaining biological development on a sustained basis in irrigated agricultural systems [20,21]. These tree species are most suitable for reforestation of marginal lands and agro-forestry systems [21]. The agro-forestry industry in Pakistan offers several advantages for rural populations, including forest timber, fuelwood, fodder, food, and incomes, significantly impacting the daily lives of rural communities [21,22].

Considering these conditions around the globe, it is worth mentioning that no appreciable research has been conducted to examine the impact of soil compaction on the growth of native agro-forest trees. Keeping in view the severity of the soil compaction problem in agro-forestry systems, the current research was enacted to investigate the negative impacts of soil compaction on different morpho-physiological traits and the ameliorative effect of agro-forest trees including *E. camaldulensis*, *A. lebbeck, V. nilotica*, and *Z. mauritiana* during their initial stages of growth against the soil compaction problem. For this research, these agro-forest tree species were selected because of their multiple uses, high timber worth, importance for fodder production, firewood purpose, and value for soil conservation so that they can be successfully grown in diverse areas with different soil textures.

## 2. Material and Methods

### 2.1. Site Description

**Figure 1:**
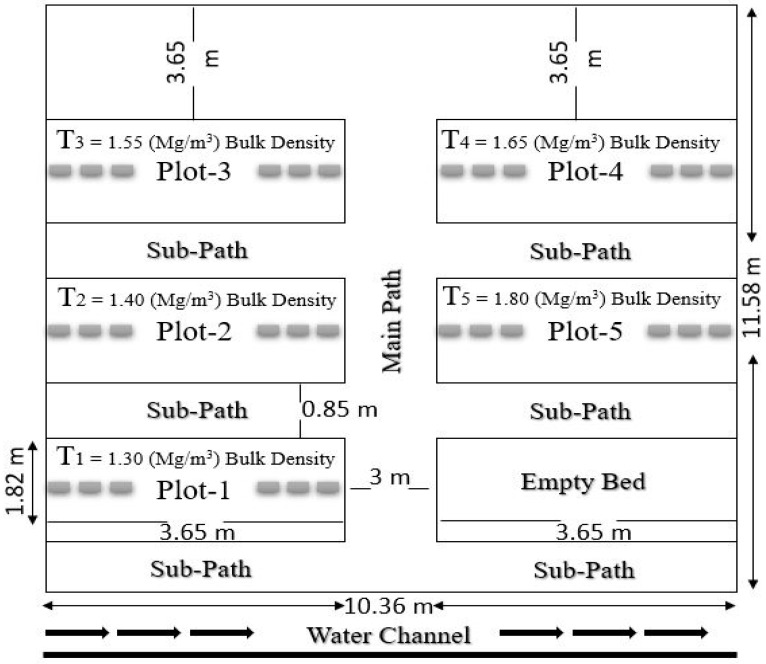
Nursery beds layout and dimensions for a controlled experiment.

A controlled condition trial was conducted in the Department of Forestry and Range Management Research Area. In this study, the impact of soil compaction was assessed on a bed nursery where four selected farm tree species (*E. camaldulensis, A. lebbeck, V. nilotica*, and *Z. mauritiana*) were raised (seeds) (Figure 1). The selected site was situated at 73.077° Longitude and 31.443° Latitude. Complete soil analysis was conducted for five randomly collected soil samples from the nursery at two depths (Table 1 and Table 2). The average minimum and maximum temperatures of January were 4.1 °C and 19.4 °C in the arid and semi-arid regions of Faisalabad. The mean annual rainfall is up to 375 mm. Rainfall is season-specific, and approximately half of the total rainfall occurs in July and August during the monsoon. Climatic data during the study period were collected from a nearby meteorological station at the University of Agriculture, Faisalabad, Pakistan (Table 3). 

### 2.2. Experimental Design

Nursery soil was loamy, having dry bulk densities around 1.3 ± 0.03 mg m^−3^ (Table 4). The experiment was conducted in earthen beds to reduce the risk of compaction variability. Five nursery beds (treatments) were prepared. The boundary was highlighted and marked using a rope, and then brick alignment was done. The soil was dragged to form sub-paths of 0.85 m wide and 3.65 m long along with the main central path, which was 3 m in width and 11.58 m in length. Sunken beds 3.65 m long and 1.82 m wide were made and lined with bricks. After removing stubbles and weeding, all the paths and beds were leveled. A manual soil compactor having 10 kg of weight from a 0.25 m height was continuously dropped and lifted to adjust the compaction levels of the prepared beds. A cone penetrometer fitted with a 25 mm diameter was used to check the compaction level quickly, in which the dial gauge provides a clear readout of penetration [23]. Then, the final bulk densities were measured for each bed (Table 4).

### 2.3. Plant Sowing and Harvesting

Seeds of four selected agro-forest tree species were sown in prepared beds. Five levels of compaction were taken, i.e., T1 (1.30 BD) mg m^−3^, T2 (1.40 BD) mg m^−3^, T3 (1.55 BD) mg m^−3^, T4 (1.65 BD) mg m^−3^, and T5 (1.80 BD) mg m^−3^ as given in (Figure 1). Sixty seeds were used per species per bed under one treatment. However, six replications were used within each bed to achieve the desired numbers. Different cultural practices (weeding, cleaning, irrigation, etc.) were carried out as required. Before sowing seeds, flood irrigation and then sprinkling were applied until germination. After complete germination, light flood irrigation was carried out again. Data regarding the following parameters were recorded regularly. 

### 2.4. Morphological Parameters Used in the Study

Plants were harvested (20 plants/specie/treatment), and root length, shoot length, diameter at root collar, root-fresh weight, root-dry weight, shoot-fresh weight, shoot-dry weight, root–shoot ratio, germination percentage, survival percentage, no. of leaves, no. of branches, and leaf area were measured. Immediately after harvest (after about 7–8 months), the plants, root, and shoot length were measured with measuring tape, while the vernier caliper was used to determine the diameter of the root collar. After measuring root and shoot fresh weight, plant samples were put into the oven (DGH-9202 series thermal electric thermostat drying oven) at 75 °C for 24 h for the drying process, then the biomass of root and shoot samples was weighed with an electrical balance (electronic scale JJ3000B, Shanghai Shenguang Instrument Co.,Ltd, Shanghai, China). Germination and survival percentage was recorded regularly. Leaf area was measured using a leaf area meter (YAXIN-1241/CI-20-CID).

### 2.5. Physiological Parameters Used in the Study

Net photosynthetic rate, transpiration rate, stomatal conductance, internal carbon dioxide concentration, and photosynthetic water use efficiency were determined using the IRGA, LCA-4, Analytical Development Company, Hoddesdon, England [24,25,26].

### 2.6. Statistical Analysis

Analysis of variance (two factorial under RCBD) for the effect of species, treatments, and interaction between them was carried out on different morphophysiological traits and biomass distribution. All statistical analyses were performed using the SPSS Statistical Package (SPSS 17.0, SPSS Ins., Chicago, IL, USA). Results were statistically analyzed using a *p* < 0.05 significance level. Graph Pad Prism 9 software was used to make graphs.

## 3. Results 

### 3.1. Root Length and Shoot Length

**Figure 2:**
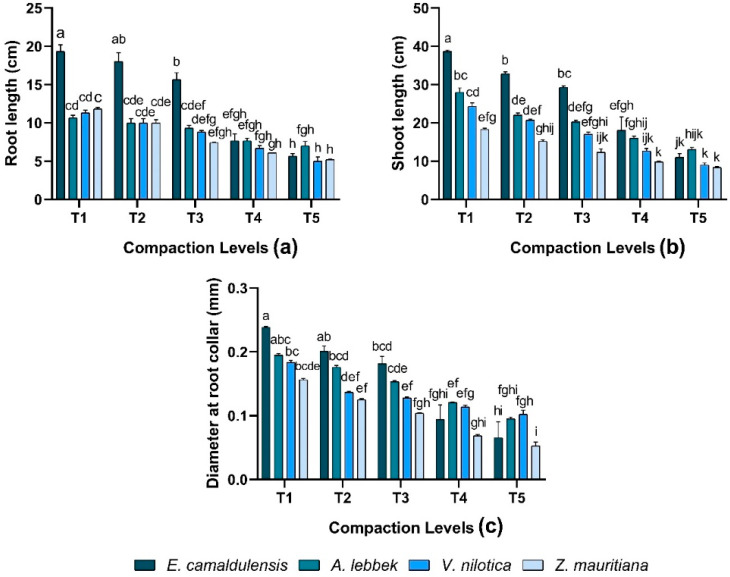
Effect of soil compaction on root length (cm), shoot length (cm), and diameter at root collar (mm) of different agro-forest tree species (**a**–**c**). Note: Values are mean ± SE. For root length, shoot length, and diameter at root collar, there were significant differences (*p* < 0.05) by the different lowercase letter(s) among *E. camaldulensis, A. lebbeck, V. nilotica*, and *Z. mauritiana* in different compaction levels.

A significant difference was observed in root length (RL) against different soil compaction levels (*p* < 0.01). The interaction between species and treatment observed significant (*p* < 0.01) variations. In *E. camaldulensis, Z. mauritiana, V. nilotica,* and *A. lebbeck,* maximum RL 19.33 ± 0.88, 11.83 ± 0.16, 11.33 ± 0.33, and 10.67 ± 0.33 cm was observed in T1 treatment while T5 treatment observed lower RL in all species (Figure 2a). Shoot length (SL) varied significantly (*p* < 0.01) in all species; meanwhile, there were significant differences found in the interaction between species and treatments (*p* < 0.01). SL observed was greater in *E. camaldulensis* 38.66 ± 0.33 cm while *Z. mauritiana* 18.33 ± 0.33 cm produced shorter stems under T1 treatment than *E. camaldulensis*. Overall, *E. camaldulensis* exhibited higher RL and SL compared to other species except for T4 and T5. Plant morphological growth decreased as the compaction levels increased (Figure 2b).

### 3.2. Diameter at the Root Collar

In terms of diameter at root collar (DRC), all species were found to vary significantly (*p* < 0.01) in response to different soil compaction levels, and the interaction between species and treatment was also significant (*p* < 0.01). DRC was revealed in all species with the trend of *E. camaldulensis* > *A. lebbeck* > *V. nilotica* > *Z. mauritiana* under similar compaction levels except T4 and T5. Results demonstrated that DRC increased as the compaction level decreased in all species (Figure 2c).

### 3.3. Biomass Distribution

**Figure 3:**
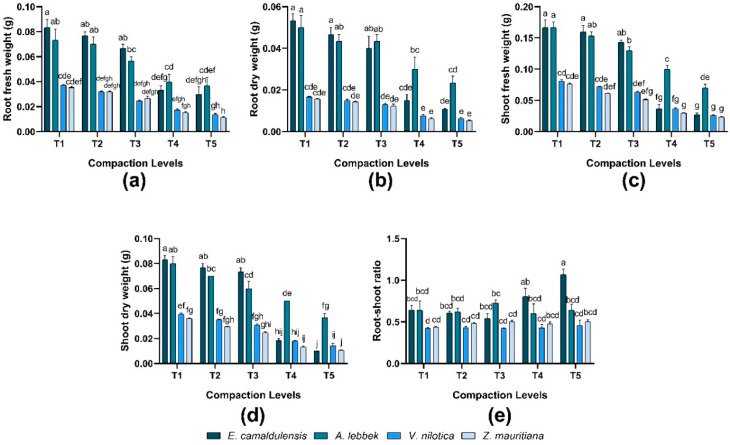
Effect of soil compaction on root and shoot weights (fresh and dry) g and root–shoot ratio (dry weights) of different agro-forest tree species (**a**–**e**). Note: Values are mean ± SE. For root and shoot (fresh and dry weights) and root–shoot ratio, there were significant differences (*p* < 0.05) by the different lowercase letter(s) among *E. camaldulensis, A. lebbeck, V. nilotica*, and *Z. mauritiana* in different compaction levels.

Root and shoot (fresh and dry weight) were significantly different in all species, while the interaction between species and treatment also revealed significant (*p* < 0.01) in response to different compaction levels. *E. camaldulensis* observed greater root biomass (T1, T2, and T3), whereas *Z. mauritiana* exhibited lower biomass under different compaction levels. In terms of shoot biomass, the same trend was found in root biomass. Consequently, the root and shoot biomass of all species decreased as the compaction level increased. In terms of root–shoot ratio, *E. camaldulensis* was found to be greater than other species. The root–shoot ratio was analyzed directly proportional to compaction levels except for *V. nilotica* and *Z. mauritiana* (Figure 3). However, the interaction between species and treatment showed significant behavior (*p* = 0.001).

### 3.4. Plant Organs Distribution

According to the statistical analysis, the growth of different plant organs (leaves, branches, leaf area) was observed to be significant (*p* < 0.01) in all species. In contrast, the interaction between species and treatment revealed significance in leaves (*p* = 0.05), branches (*p* < 0.01), and leaf area (*p* < 0.01) in response to soil compaction. Results concluded that *Z. mauritiana* recorded a maximum no. of leaves, while the larger leaf area and higher no. of branches were observed in *E. camaldulensis* under treatment (T1) compared to other species. All plant organs found a decline in growth in response to high compaction levels (T5), as shown in (Table 5). 

### 3.5. Physiological Plant Trait

**Figure 4:**
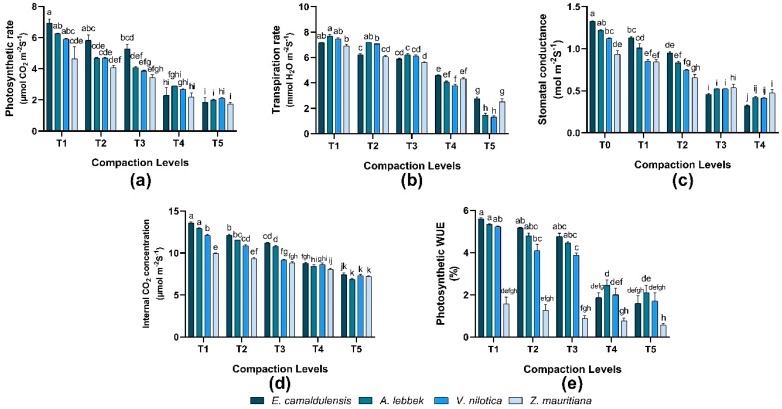
Effect of soil compaction on plant physiological traits of different agro-forest tree species (**a**–**e**). Note: Values are mean ± SE. For physiological traits, there were significant differences (*p* < 0.05) in the different lowercase letter(s) among *E. camaldulensis, A. lebbeck, V. nilotica*, and *Z. mauritiana* in different compaction levels.

Net photosynthetic rate, transpiration rate, stomatal conductance, internal CO_2_ concentration, and photosynthetic water use efficiency varied significantly (*p* < 0.01) in all species, whereas the interaction between species and treatments was observed to be significant (*p* = 0.01). *E. camaldulensis* indicated a maximum photosynthetic rate of 6.92 ± 0.27 µmol CO_2_ m^−2^S^−1^. Consequently, the transpiration rate was higher in *A. lebbeck* 7.7 ± 0.11 mmol H_2_O m^−2^S^−1^. *E. camaldulensis* showed higher stomatal conductance of 1.32 ± 0.01 mol m^−2^S^−1^, an internal CO_2_ concentration of 13.6 ± 0.11 µmol m^−2^S^−1,^ and photosynthetic WUE of 5.6 ± 0.05% was recorded under lower compaction. However, a decline in physiological traits was found under (T5) treatment (high compaction) in all species (Figure 4).

### 3.6. Germination Rate and Survival Percentage

**Figure 5:**
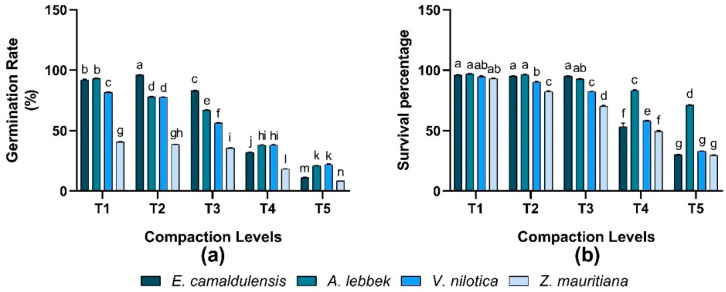
Effect of soil compaction on plant germination rate and survival percentage (%) of different agro-forest tree species (**a**,**b**). Note: Values are mean ± SE. For germination rate and survival percentage, there were significant differences (*p* < 0.05) by the different lowercase letter(s) among *E. camaldulensis, A. lebbeck, V. nilotica*, and *Z. mauritiana* in different compaction levels.

It was observed that the response of the germination rate of all species was significant, and the interaction between species and treatment was also significantly varied (*p* < 0.01) in all treatments. Maximum germination of 96% was observed in *E. camaldulensis* under (T2) treatment, while 93% in *A. lebbeck*, 81.67% in *V. nilotica,* and 40.67% in *Z. mauritiana* in response to T1 treatment. The minimum germination rate of all species was found in (T5) treatment (Figure 5). All species’ response of survival percentage was significantly different in different imposed treatments. *A. lebbeck* found a greater survival percentage of 97% when treatment (T1) was applied to the growing media (Figure 5).

## 4. Discussion

The current study assessed the growth response of four major agro-forest species (*E. camaldulensis, A. lebbeck, V. nilotica,* and *Z. mauritiana*) against soil compaction. The growth of selected species was strongly affected by increasing soil compaction level, which supported the concept that increasing bulk density decreases all measured morpho-physiological traits of the seedlings. Increasing soil bulk density particularly influences root development [12,27]. Penetration of roots improves pore continuity, lowers bulk density, and enhances soil aeration, whereas biological waste provides minerals that will improve soil fertility [28,29].

*E. camaldulensis* was the better-ranked species that exhibited maximum root and shoot growth (at < 1.65 mg m^−3^), whereas *Z. mauritiana* showed minimum growth under all applied compaction levels (Figure 2). Increased soil resistance can enhance plant stress by reducing growth performance (plant biomass, root and shoot length, and diameter) (Figure 2 and Figure 3) and boosting seedling mortality [30,31,32,33]. Roots in compacted soils can be thicker and shorter, although, in good conditions, they can grow largely in width to maintain overground development and have the potential to tolerate increased penetration resistance [34,35]. Compaction can influence primary root growth immediately after germination [10].

Root and shoot biomass growth response of all species was revealed better against control treatment, which worsened with the increased level of soil compaction [6,36,37]. As shown in Figure 3, *E. camaldulensis* indicated greater root and shoot biomass under no compaction (1.30 mg m^−3^) or light compaction (1.55 mg m^−3^). In comparison, a sudden decline was found in response to high compaction levels (1.80 mg m^−3^) as compared to other species [38]. Compaction can seriously damage the emergence and growth of the seedlings and can severely affect the root system of plants by reducing their competence to retrieve nutrients in the soil but had a moderate effect on shoot growth. When soils are extremely compacted, the movement of ions, oxygen, microorganisms, and water in the soil is decreased because macropores turn into micropores which strictly confine the root and shoot growth [6,10,39]. According to the studies of Alameda and Villar [40], an increase in soil strength can affect the architecture and overall biomass production of roots of woody plants due to the restricted availability of nutrients [38,41]. This ultimately results in poor leaf growth, decreased photosynthetic and transpiration rate, poor stomatal conductance, and poor water use efficiency [39,40,42]. 

The maximum root-to-shoot ratio was observed for *E. camaldulensis* in highly compacted soil (Figure 3). It was considered that the root-to-shoot ratio response to soil compaction is inappropriate to evaluate its overall impact on plant growth because it depends on soil type, water contents, and light conditions (1.55 mg m^−3^) [27,41]. *E. camaldulensis* produced a maximum leaf area and a number of branches, although a greater number of leaves were observed in *Z. mauritiana*, under control conditions and at a light compaction level (1.55 mg m^−3^) (Table 5). These results align with the findings of [37,43], who suggested that intense compaction results in poor growth of plant parts. However, *E. camaldulensis* showed a comparatively high germination percentage at light compaction compared to others [44]. 

A meta-analysis by Mariotti et al. [42] showed that higher compaction reduced the photosynthesis of the leaves. This relates to our findings; the maximum rate of photosynthesis was recorded in *E. camaldulensis* under control conditions, whereas reduction in photosynthesis was influenced by the high intensity of compaction level (Figure 4a). Compaction has been considered to limit the availability of water and nutrients and diminution in the leaf photosynthesis of plants [17]. Soil compaction also restricts root hydraulic conductivity, which plays a major role in water absorption. A considerable decrease in transpiration was found to accompany lower photosynthesis [45]. The current study exhibited a high transpiration rate in *A. lebbek*, and the decline was seen in *Z. mauritiana* at control conditions. However, a decline in transpiration in all species was found at a high compaction level (Figure 4b). These outcomes recommend that soil compaction shortened main roots and negatively affected plant physiology and drought stress tolerance of the seedlings. As in compacted soils, stomatal closure results in a reduction in the availability of CO_2_ in the mesophyll [42,46].

Moreover, excess light energy and reactive oxygen species formation (ROS) might be induced and inflict further damage to photosynthesis [33]. A decrease in chlorophyll fluorescence characteristics justified this prediction in several experiments [33,47]. In our findings, higher stomatal conductance, internal CO_2_ concentration, and Photosynthetic WUE were recorded in *E. camaldulensis* followed by the remaining species in control conditions (Figure 4c–e); however, highly compacted soil exposed a decline in mentioned physiological attributes. Different growth response (regarding morphology and physiology) of species mentioned above exactly matches with the findings of [39], which stated that different plant species have different tolerance level against soil compaction-compacted agricultural lands may limit the growth and survival of only a few species and vice versa. But in developing countries (like Pakistan), resources are scarce to avoid subsoil compaction to address such issues.

The compaction of the soil can generally influence the soil ecology negatively [17]. However, soil compaction causes physical changes that may not lead to significant changes in nutrient availability to plants [48]. This may be due to greater root-to-soil contact for nutrients; however, compaction usually reduces the lengthening of the primary root for nutrient absorption [49]. The nutritional availability of plants also refers to soil microbial activity. Due to the compaction of soils, unfavorable soil conditions might alter the entire structures of the bacterial community [50,51]. Different results of the current study may be due to differences in soil texture and its ability to retain water. Loamy to clay soil was used that can become compacted easily [31,52], which may have resulted in oxygen-deficit soil with low moisture contents and porosity. Since the overall porosity of the soil decreases as a result of soil compaction, the water penetration rate in the soil may be used to determine soil compaction [53]. Water penetration is weaker in extremely compacted soil than in uncompacted soil in the same soil type [54]. Moreover, moderate compaction of coarse-textured soil generally improves root contact with soil, which helps in better nutrient absorption [6,55].

## 5. Conclusions

This study measured the extent to which the morphological and physiological attributes of the agro-forestry tree species decreased after manually induced soil compaction and expressed a rise in bulk density. For evaluating compaction, soil bulk densities were used as an index. It was concluded that soil compaction has a generally detrimental influence on growth. It can be managed by improving the accumulation of organic matter and mechanization to enhance plant growth. In the future, this issue can be re-addressed in areas with serious vehicular traffic on other soil types.

## Figures and Tables

**Table 1:** Texture of nursery soil.

	Sand (%)	Silt (%)	Clay (%)
**0–15 cm (Loam)**	40 ± 3	45 ± 3	15 ± 2
**15–30 cm (Sandy loam)**	69 ± 5	18.5 ± 2	12.5 ± 2

**Table 2:** Chemical properties of nursery soil.

	pH	* EC (dSm^−1^)	** TSS (ppm)	Nitrogen (%)	Phosphorous (ppm)	Potassium (ppm)	Organic Matter (%)
0–15 cm	8.0 ± 0.01	1.68 ± 0.1	1176 ± 35	0.07 ± 0.005	3.9 ± 0.1	280 ± 5	1.54 ± 0.05
15–30 cm	8.2 ± 0.02	1.35 ± 0.1	1236 ± 10	0.05 ± 0.005	9.8 ± 0.2	250 ± 5	0.91 ± 0.02

* EC = Electrical conductivity; ** TSS = Total soluble salts.

**Table 3:** Climatic conditions of the experimental site.

Months /Years	Temperature (°C)			* R.H. (%age)	Rainfall (mm)	Sunshine Duration (Hours)	** Pan Evap. (mm)	*** Evap. Transp. (mm)	Wind Speed (km/h)
	Max.	Min.	Avg.						
Nov/2018	27.0	12.4	19.7	74.6	0.6	6.9	1.9	1.4	3.4
Dec/2018	21.7	6.5	14.1	81.5	0.7	6.9	1.0	0.9	2.9
Jan/2019	19.2	7.0	13.1	80.7	18.0	5.4	1.2	0.8	4.8
Feb/2019	20.3	9.1	14.7	79.0	64.2	6.7	1.6	1.1	4.4
Mar/2019	26.0	13.8	19.9	68.5	55.7	8.9	3.0	2.1	4.8
Apr/2019	35.0	20.6	27.8	42.5	31.2	9.0	5.4	3.8	5.0
May/2019	39.0	23.9	31.4	46.5	39.1	10.1	6.8	4.8	5.0
Jun/2019	42.4	27.4	34.9	47.8	35.5	10.1	8.5	6.0	5.7
Jul/2019	38.0	28.0	33.0	62.7	102.8	7.4	5.5	3.9	5.8
Aug/2019	38.0	28.5	33.2	72.5	80.9	7.7	4.5	3.2	4.3

* R.H. = Relative humidity; ** Pan Evap = Pan evaporation; *** Evap Transp. = Evapotranspiration.

**Table 4:** Bulk densities (controlled, 10%, 20%, 30%, and 40%, established in a nursery for experimentation).

Beds	Bed-1 (T1) Controlled	Bed-2 (T2) (10%)	Bed-3 (T3) (20%)	Bed-4 (T4) (30%)	Bed-5 (T5) (40%)
**B. Densities (** **mg m^−3^** **)**	1.3 ± 0.03	1.40 ± 0.05	1.55 ± 0.04	1.65 ± 0.08	1.8 ± 0.1

**Table 5:** Effect of soil compaction on plant organs distribution of different agro-forest tree species.

		T1	T2	T3	T4	T5
**No. of Leaves**	*E. camaldulensis* *A. lebbeck* *V. nilotica* *Z. mauritiana*	65.67 ± 1.76	59.00 ± 1.15	54.00 ± 1.00	36.33 ± 3.28	26.67 ± 2.85
		56.00 ± 1.00	49.33 ± 1.45	41.33 ± 3.28	24.00 ± 1.00	16.00 ± 1.53
		47.33 ± 1.76	43.33 ± 0.88	35.67 ± 1.86	25.00 ± 2.31	19.33 ± 0.33
		69.66 ± 0.88	56.33 ± 2.33	49.00 ± 2.65	42.00 ± 0.57	28.33 ± 5.70
**No. of Branches**	*E. camaldulensis* *A. lebbeck* *V. nilotica* *Z. mauritiana*	20.67 ± 1.33	16.00 ± 1.15	14.33 ± 0.66	5.66 ± 0.66	4.66 ± 0.33
		12.00 ± 0.57	10.33 ± 0.33	9.00 ± 0.57	7.66 ± 0.33	5.66 ± 0.33
		6.00 ± 0.57	5.33 ± 0.33	5.00 ± 0.57	3.33 ± 0.33	2.33 ± 0.33
		17.33 ± 0.33	15.33 ± 0.33	12.33 ± 1.20	6.00 ± 1.73	3.66 ± 0.66
**Leaf Area (mm^2^)**	*E. camaldulensis* *A. lebbeck* *V. nilotica* *Z. mauritiana*	936.6 ± 1.95	866.5 ± 2.11	822.5 ± 1.57	617.6 ± 33.0	479.9 ± 34.6
		76.40 ± 0.72	70.30 ± 0.70	62.26 ± 0.66	43.73 ± 0.38	33.13 ± 2.50
		6.20 ± 0.05	5.80 ± 0.05	5.76 ± 0.03	5.53 ± 0.03	5.48 ± 0.04
		62.37 ± 1.27	61.96 ± 0.29	60.53 ± 0.20	59.06 ± 0.52	58.26 ± 0.08

## Data Availability

Data can be provided by formal request to corresponding author (MHUR).

## Abbreviations

*Eucalyptus camaldulensis*	*E. camaldulensis*
*Albizia lebbeck*	*A. lebbeck*
*Vachellia nilotica*	*V. nilotica*
*Zyziphus mauritiana*	*Z. mauritiana*
Bulk Density	BD
Root length	RL
Shoot length	SL
Diameter at root collar	DRC
Photosynthetic Water Use Efficiency	Photosynthetic WUE
